# Modeling gene sequences over time in 2009 H1N1 Influenza A Virus populations

**DOI:** 10.1186/1743-422X-6-215

**Published:** 2009-12-04

**Authors:** Natalia Goñi, Alvaro Fajardo, Gonzalo Moratorio, Rodney Colina, Juan Cristina

**Affiliations:** 1 Laboratorio de Virología Molecular, Centro de Investigaciones Nucleares, Facultad de Ciencias, Igua 4225, 11400 Montevideo, Uruguay

## Abstract

**Background:**

A sudden emergence of Influenza A Virus (IAV) infections with a new pandemic H1N1 IAV is taking place since April of 2009. In order to gain insight into the mode of evolution of these new H1N1 strains, we performed a Bayesian coalescent Markov chain Monte Carlo (MCMC) analysis of full-length neuraminidase (NA) gene sequences of 62 H1N1 IAV strains (isolated from March 30^th ^to by July 28^th^, 2009).

**Results:**

The results of these studies revealed that the expansion population growth model was the best to fit the sequence data. A mean of evolutionary change of 7.84 × 10^-3 ^nucleotide substitutions per site per year (s/s/y) was obtained for the NA gene. A significant contribution of first codon position to this mean rate was observed. Maximum clade credibility trees revealed a rapid diversification of NA genes in different genetic lineages, all of them containing Oseltamivir-resistant viruses of very recent emergence. Mapping of naturally occurring amino acid substitutions in the NA protein from 2009 H1N1 IAV circulating in 62 different patients revealed that substitutions are distributed all around the surface of the molecule, leaving the hydrophobic core and the catalytic site essentially untouched.

**Conclusion:**

High evolutionary rates and fast population growth have contributed to the initial transmission dynamics of 2009 H1N1 IAV. Naturally occurring substitutions are preferentially located at the protein surface and do not interfere with the NA active site. Antigenic regions relevant for vaccine development can differ from previous vaccine strains and vary among patients.

## Background

Influenza A virus (IAV) is a member of the family *Orthomyxoviridae *and contains eight segments of a single-stranded RNA genome with negative polarity [1]. IAV causes 300,000-500,000 deaths worldwide each year, and in pandemic years, this number can increase to 1 million (in 1957-1958) or as high as 50 million, as was seen in 1918-1919 [2]. Unlike most pathogens where exposure leads to lasting immunity in the host, IAV presents a moving antigenic target [3], evading specific immunity triggered by previous infections. This process, called antigenic drift, is the result of the selective fixation of mutations in the gene encoding the hemagglutinin (HA) protein, the major target for the host immune response [4]. Variants that best escape the host immune response are thought to have a significant reproductive advantage [5].

Another process, called antigenic shift, is also considered a major force in the evolution of IAV [4,5]. Antigenic shift occurs when the virus acquires an HA of a different IAV subtype via reassortment of one or more gene segments and is thought to be the basis for the more devastating influenza pandemics that occurred several times in the last century [6]. New IAV pandemics may emerge through reassortation with strains from swine or avian reservoirs [7].

There have been three pandemics in the last hundred years: in 1918 (H1N1 subtype) [8], 1957 (H2N2 subtype) [9], and in 1968 (H3N2 subtype) [10]. During each of these pandemics, the new virus drove the previous pandemic subtype out of circulation [3]. In 1977, the H1N1 subtype reappeared, and has been co-circulating with H3N2 since then [11,12].

IAV H3N2 viruses have been the predominant strains during the last 20 years, with the exception of the 1988-1989 and 2000-2001 seasons where H1N1 infections dominated [13].

A sudden emergence of IAV infections with new H1N1 strains of pandemic potential is taking place since April of 2009, starting in Mexico and spreading to several other countries around the world [14]. The World Health Organization (WHO) has raised the Influenza pandemic alert to the maximum level 6 [15].

Oseltamivir phosphate is a prodrug of oseltamivir carboxylate, a highly specific inhibitor of IAV neuraminidases. Oseltamivir carboxylate binds to highly conserved, essential amino acids in the catalytic site of neuraminidase (NA), preventing virus release from infected cells and subsequent virus spread [16]. An amino acid substitution at position 275 (H275Y) of the NA protein has been associated to resistance to Oseltamivir [17].

Initial testing of the 2009 pandemic H1N1 IAV strains found the viruses to be susceptible to neuraminidase inhibitors (oseltamivir and zanamivir).

Detailed studies on the mode of evolution of these new H1N1 IAV strains are extremely important for our understanding of the molecular mechanisms involved in the emergence, spread and resistance of new H1N1 IAV strains of pandemic potential. In order gain insight into these matters, we have performed a Bayesian coalescent Markov chain Monte Carlo analysis of full-length NA gene sequences of 62 emerging 2009 H1N1 IAV strains (isolated from March 30^th ^to July 28^th^, 2009). The results of these studies revealed high rate of evolutionary change of NA genes, fast expansion of the H1N1 IAV populations and emergence of anti-viral resistant viruses. Naturally occurring amino acid substitutions in the NA of H1N1 IAV strains circulating in 62 different patients preferentially located at the protein surface and do not interfere with the NA active site.

## Methods

### Neuraminidase sequences

Full-length NA sequences from the 2009 emerging H1N1 IAV strains, were obtained from The Influenza Virus Resource at the National Center for Biotechnological Information [18]. For strain names, dates of isolation and accession numbers see Table S1, Additional file 1.

### Sequence alignment

NA sequences were aligned using the MUSCLE program [19].

### Evolutionary Model analysis

Once aligned, the FindModel program [20] was used to identify the optimal evolutionary model that best fitted our sequence dataset. Akaike Information Criteria revealed that the General Time Reversible (GTR) model was the best fit to the data (Table S2, Additional file 2).

### Recombination Detection Tests

To test whether a recombination event occurred on any of the sequences included in these studies, two different approaches implemented in the SimPlot program [21] were used: (1) a sliding window analysis of distances and (2) the bootscanning [22]. No recombinant strains were found in the datasets (not shown).

### Bayesian Coalescent Inference Studies

The evolutionary rate and mode of evolution of the newly emerging 2009 H1N1 IAV strains were determined using a coalescent Bayesian Markov chain Monte Carlo (MCMC) approach as implemented in the BEAST package [23]. Sixty-two full-length NA gene sequences were included in these analyses. For names, accession numbers and date of isolation of strains included in these studies, see Table S1, Additional file 1,. Using the GTR model, 60 million steps of MCMC and dates introduced by day of isolation, different population dynamic models were tested (constant population size, exponential population growth, expansion population growth, logistic population growth and Bayesian Skyline). Statistical uncertainty in the data was reflected by the 95% highest probability density (HPD) values. Results were examined using the TRACER program from the BEAST package [24]. Convergence was assessed with ESS (Effective Sample Size) values, after a burning of 6 million steps. Maximum clade credibility trees were generated using Tree Annotator from the BEAST package and the FigTree v1.2.2 (available at: http://tree.bio.ed.ac.uk/) was used for the visualization of the annotated trees.

## Results

### Modelling gene sequences changes over time in NA gene of 2009 H1N1 emerging strains

In order to gain insight into the evolutionary rate and mode of evolution of 2009 H1N1 IAV strains, we used a Bayesian Markov Chain Montecarlo (MCMC) approach to analyze 62 full-length NA gene sequences from 2009 H1N1 IAV strains isolated from March 30^th ^to July 28^th^, 2009 (for strains names, accession numbers and dates of isolation, see Table S1, Additional file 1,).

Using the GTR model and 60 million steps of MCMC, different population dynamics models were tested (constant population size, exponential population growth, expansion population growth, logistic population growth and Bayesian skyline). Statistical uncertainty in the data was reflected by the 95% highest probability density (HDP) values. Convergence was assessed with Effective Sample Size (ESS) values, after a burning of 6 million steps. Comparison of the values obtained for marginal likelihoods as well as ESS of these models revealed that the Expansion Population Growth model was the best to fit the data.

The results shown in Table 1 are the outcome of the analysis for 60 million steps of the MCMC, using the GTR model, a relaxed clock [24] and the Expansion Population Growth model [25].

As can be seen in Table 1, our results suggest that the NA gene of the 2009 H1N1 emerging IAV strains evolved from ancestors that existed around August 17^th^, 2008. This is in agreement with previous results situating the most recent common ancestor (MRCA) for the NA gene of 2009 H1N1 IAV around August 8^th^, 2008 [26].

When the GTR model is used, a mean of 7.84 × 10^-3 ^nucleotide substitutions per site per year (s/s/y) was obtained for the NA gene (Table 1). This rate is roughly comparable to previous estimations of IAV NA evolutionary rates (3.6 × 10^-3 ^s/s/y) [26]. Interestingly, a significant contribution of the first codon position to the evolutionary rate was also found (Table 1). Moreover, an important expansion growth rate was observed (see Table 1).

**Table 1:** Bayesian coalescent inference of full-length NA sequences from 2009 H1N1 Influenza A virus strains.

Group	Parameter	Value^a^	HPD^b^	ESS^c^
62 NA sequences	Log likelihood	-2601.35	-2616.89 to -2586.07	1401.12
	Mean Rate^d^	7.84 × 10^-3^	7.59 × 10^-3 ^to 1.43 × 10^-2^	126.75
	Codon 1^e^	0.97	0.67 to 0.95	29387.19
	Codon 2	0.51	0.25 to 0.79	40307.00
	Codon 3	1.51	1.17 to 1.86	26291.08
	Expansion Growth Rate^f^	66.43	0.38 to 503.70	206.45
	Root age (days)	324.99	130.84 to 644.01	163.42
	MRCA^g^	August 17^th^, 2008	September 27^th^, 2007 to March 9^th^, 2009.	

^a^In all cases, the mean values are shown. ^b ^HPD, high probability density values. ^c ^ESS, effective sample size. ^d ^Mean rate was calculated in substitutions/site/day and transformed to substitution/site/year. ^e ^Contribution of each codon position to the mean rate. ^f ^Expansion Growth Rate was calculated in number of new infections/individual/day and transformed to number of new infections/individual/year. ^g ^MRCA, day of the Most Common Recent Ancestor.

### Phylogenetic tree analysis of NA genes from 2009 H1N1 IAV strains

**Figure 1:**
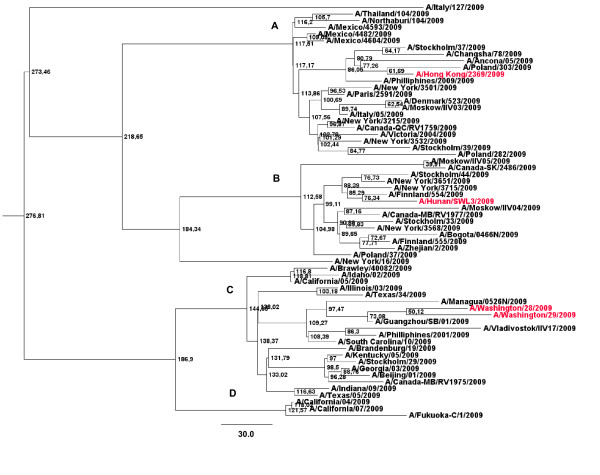
**Bayesian MCMC phylogenetic tree analysis of 62 NA genes from 2009 H1N1 IAV strains**. A maximum credibility clade obtained using the GTR model, the expansion population growth model and a relaxed clock (uncorrelated exponential) is shown. Strains in the tree are shown by name. Main genetic sub-branches are indicated by capital letters (A through D). Node ages are shown in days at the nodes of the tree. The tree is rooted to theirMRCA. Bar at the bottom of the tree show time in days. Strains carrying the H275Y, that confers resistance to Oseltamivir, are shown in red.

To study the phylogenetic relations among the NA genes from the 62 H1N1 IAV strains enrolled in these studies, maximum clade credibility trees were generated using software from the BEAST package [23]. The results of these studies are shown in Figure 1.

As it can be seen in the figure, different genetic sub-branches can be observed. Interestingly, Oseltamivir-resistant viruses can be observed in all main genetic sub-branches. These viruses are situated on the tip of the trees suggesting a recent emergence from the 2009 H1N1 IAV populations (see Figure 1). This is in agreement with the initial studies revealing that 2009 H1N1 IAV strains were susceptible to Oseltamivir and the recent selection of resistant viruses from these viral populations [17].

### Mapping of positive-selected and co-evolving sites in a 3D NA protein model

**Figure 2:**
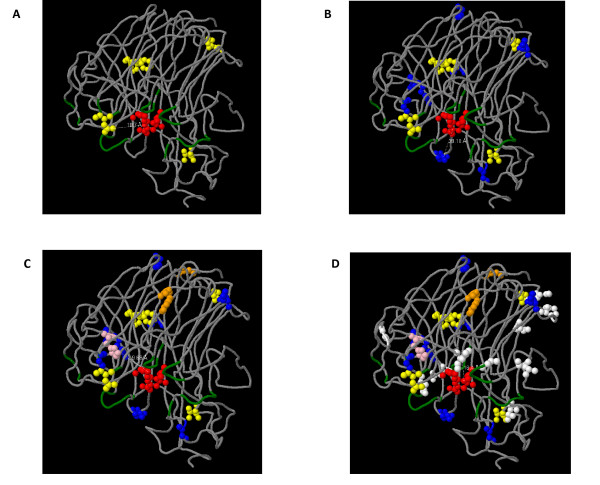
**Mapping of naturally occurring amino acid substitutions in a NA protein 3D structure**. The 3D structure model of the NA protein from 2009 H1N1 IAV shown in the figure was obtained by Mauer-Stroh et al. [27] (Bioinformatic Institute, A*STAR's Biomedical Sciences Institutes, Singapore). Oseltamivir atoms are shown in red. Antibodies binding sites are shown in green. Position 275, where substitution H275Y confers resistance to Oseltamivir, is shown in pink. The substitutions found among strains isolated during 30, 60, 90 (where viruses with H275Y substitution also arise) and 119 days (from March 30^th^, 2009) are shown in yellow, blue, orange and white in A through D, respectively. Dotted white lines show distances in Å.

An homology-based 3D structure model of the NA protein of 2009 H1N1 IAV strains have been very recently obtained [27] (available at http://mendel.bii.a-star.edu.sg/SEQUENCES/H1N1/). In order to observe if the amino acids substitutions naturally occurring in the NA genes of the 62 H1N1 IAV studied were associated to previously identified antigenic regions or the active site of the NA protein (being the latter the binding cavity of Oseltamivir and other NA inhibitors drugs), we mapped all substitutions found in NA proteins of all IAV enrolled in these studies in a temporal order, according to the date of isolation of each strain. The results of these studies are shown in Figure 2.

As it can be seen in the figure, no substitution was found to be related to the active site of the NA protein (see Figure 2). Importantly, none of the substitutions found in our dataset appears sufficiently close to affect the drug binding pocket (see Figure 2).

## Discussion

The antigenic variability of IAV is the basis for the recurring epidemics each year [28]. IAV presents a moving antigenic target, evading immunity triggered by previous infections. For these reasons, efforts to characterize epidemic variants [29] are deemed important for improving influenza vaccine formulation, since the closer the vaccine strain is to the dominant variant, the more effective the vaccine [30].

A sudden emergence of new H1N1 IAV of swine origin is taking place since April of 2009 [15]. This pandemic started in Mexico and it is currently spreading to all regions of the world [14]. On June 11^th^, the WHO officially raised the phase of pandemic alert to level 6. As of July 19^th^, 137,232 cases of the 2009 H1N1 IAV emerging strains have been officially confirmed in 142 countries [31].

Different approaches have been extremely useful in increasing our understanding of the spatial-temporal transmission dynamics of influenza. They have also provided assistance in evaluating the potential severity of IAV pandemics, where severity was defined by the value of the Basic Reproduction Number (*R*_0_) [32].

The *R*_0 _for novel influenza A (H1N1) has recently been estimated to be between 1.4 and 1.6 [33], revealing an important expansion of this IAV population. Fortunately, this value is below values of *R*_0 _estimated for the 1918-1919 pandemic strain (mean *R*_0_~2, range 1.4 to 2.8) [32].

These results are in agreement with the results found in this work using a Bayesian coalescent MCMC approach (see Table 1). A high expansion growth rate (66.43 new infections/individual/year) was achieved, particularly considering the short period of time studied (March 30^th ^to July 28^th^, 2009). These results suggest that the pandemic caused by the 2009 H1N1 IAV will continue its expansion phase at a significant rate.

We estimated that the NA of the 2009 H1N1 IAV evolved from ancestors that existed around August 17^th^, 2008 (Table 1). Interestingly, this date is in agreement with first estimations of the MRCA for that gene of these pandemic strains (August 8^th^, 2008) [26]. This result suggests that the NA gene segment of these viruses were presumably circulating in the swine reservoir before emerging into the human population, in agreement with recent results [34].

The first estimation of evolutionary rate for the NA gene of the 2009 H1N1 IAV strains established a rate of 3.65 × 10^-3 ^[26]. In this study, a mean evolutionary rate of 7.84 × 10^-3 ^s/s/y was obtained (see Table 1). Although not entirely dissimilar rates are found, the possible differences among the two estimations may be due to the fact that the first estimations were carried out at the beginning of the pandemic outbreak, where only 30 NA sequences isolated over a shorter time span (from March to May) were available [26]. In this work, 62 full-length NA sequences, isolated from March to the end of July, were employed (see Table S1, Additional file 1). Importantly, a contribution of first codon position of 0.97 (from a total of 3.0) to the mean evolutionary rate was found (Table 1). This speaks of a comparatively higher contribution of non-synonymous substitutions to the mean substitution rate. This result is in agreement with previous reports showing a comparatively higher non-synonymous to synonymous (*dn/ds*) substitution rate ratio in the 2009 H1N1 IAV strains [26]. Moreover, a contribution of first codon position to main evolutionary rate like the one found in this study is significantly higher than the ones previously found in other RNA viruses, like Hepatitis A virus (0.33, VP1 gene) [35] and Noroviruses (0.55, VP1 gene) [36].

Maximum clade credibility trees revealed a rapid diversification of NA genes in at least four main phylogenetic lineages (Figure 1). Nevertheless, due to the fact that the degree of genetic variation among all strains included in these analysis is roughly low (with a maximum degree of variation of 0.64%), more studies will be needed to confirm these findings. Interestingly, Oseltamivir resistance was found only in the more recent samples and two of them, the A/Washington/28/2009 and the A/Washington/29/2009, appear to be phylogenetically and geographically linked (Figure 1). This finding also suggests that anti-viral resistant viruses can emerge in any genetic lineage, as a result of selection of mutant viruses from the viral population. Oseltamivir-resistant viruses are situated on the tips of the tree. This reveals a recent emergence from previously susceptible viruses (Figure 1). This result is in agreement with initial studies showing the susceptibility of the H1N1 IAV emerging strains to this drug and its widespread use to combat the spread of these viruses all around the world [17].

Mapping of substitutions found in the 62 NA proteins during the four month period covered by this study revealed that substitutions are distributed all around the surface of the molecule, leaving the hydrophobic core and the catalytic site essentially untouched (see Fig. 2). Nevertheless, strains carrying the H275Y substitution were also observed in these studies (see Figs 1 and 2). Very recent studies on the structure of the NA of mutant IAV strains carrying this substitution, revealed that the bulkier Tyr residue alters the orientation of the key Glu 277 residue [37]. On binding Oseltamivir, the conformation of the Glu 277 side chain of the wild type enzyme is altered such that it exposes a hydrophobic site with which the pentyloxy group of Oseltamivir interacts [37]. In the mutant enzyme, the bulkier Tyr residue at position 275 displaces the carboxyl group of Glu 277 into the binding site, such that it disrupts the hydrophobic pocket and causes a change in conformation of the pentyloxy substituent of Oseltamivir, with consequent reduction in affinity of binding of some 300-fold or greater [38].

Interestingly, this is not the case of Zanamivir, since the H275Y substitution causes only a small shift in the position of Glu 277, without disrupting the H-bonds between Glu 277 and the glycerol moiety of the drug [38]. This suggests that other NA inhibitors, like Zanamivir or Peramivir should still be effective against this H1N1 IAV strains. Importantly, substitutions observed in the 62 patients enrolled in this study suggest that changes at possible antigenic sites at NA protein surface may indeed occur (see Figure 2). For that reason, vaccines to previous strains or acquired immunity from previous IAV infections are expected to be less effective. More detailed studies on the 2009 H1N1 IAV evolution are extremely needed in order to select appropriate IAV vaccine strains.

## Conclusion

A coalescent Bayesian Markov Chain Montecarlo (MCMC) approach was used to analyze 62 full-length NA gene sequences from 2009 H1N1 IAV strains, isolated from March 30^th ^to July 28^th^, 2009. When the Expansion Population Growth model was employed a high rate of evolutionary change of 7.84 × 10^-3 ^s/s/y was obtained for the NA gene. Importantly, a significant contribution of the first codon position to the mean evolutionary rate was also found. Moreover, an important expansion growth rate of 66.43 new infections/individual/year was also observed. Taking these results together, high evolutionary rates and fast population growth have contributed to the initial transmission dynamics of 2009 H1N1 IAV. Naturally occurring substitutions are preferentially located at the protein surface and do not interfere with the NA active site. Antigenic regions relevant for vaccine development can differ from previous vaccine strains and vary among patients.

## Competing interests

The authors declare that they have no competing interests.

## Authors' contributions

NG and JC conceived the study. AF, GM and JC designed and performed the Bayesian coalescent studies and phylogenetic analysis. NG, AF, GM and RC contributed to the discussion and interpretation of the results. JC wrote the paper. All authors read and approved the final manuscript.

## Supplementary Material

Additional file 1 **Origins of the NA sequences from 2009 H1N1 IAV strains**. A table describing the names, date of isolation and accession numbers of all IAV strains included in this study. Click here for file

Additional file 2 **FindModel results for NA genes of 2009 H1N1 IAV strains**. A table describing the results found for different evolutionary models tested in this study. Click here for file
