# Supplementary material for: Modeling gene sequences over time in 2009 H1N1 Influenza A Virus populations
Source: Virol J. 2009 Dec 4;6:215. doi: 10.1186/1743-422X-6-215 (PMC2794274; doi:10.1186/1743-422X-6-215)
Supplement: Additional file 1 — Origins of the NA sequences from 2009 H1N1 IAV strains. A table describing the names, date of isolation and accession numbers of all IAV strains included in this study. [file 1743-422X-6-215-S1.DOC]

**Additional File 1. Origins of the NA sequences from 2009 H1N1 IAV strains.**

**________________________________________________________________________**

**Accession Number Date of Isolation Name**

________________________________________________________________________

FJ966956 2009/03/30 A/California/05/2009

|  |  |
| --- | --- |

FJ966084 2009/04/01 A/California/04/2009

|  |  |
| --- | --- |

GQ377078 2009/04/09 A/California/07/2009

GQ149670 2009/04/14 A/Mexico/4482/2009

FJ966969 2009/04/15 A/Texas/05/2009

GQ149631 2009/04/19 A/Mexico/4604/2009

GQ162173 2009/04/20 A/Mexico/4593/2009

CY043096 2009/04/21 A/Brawley/40082/2009

GQ117094 2009/04/22 A/Indiana/09/2009

GQ221815 2009/04/24 A/New York/16/2009

CY041132 2009/04/25 A/New York/3215/2009

GQ231980 2009/04/25 A/Mexico/47N/2009

GQ377059 2009/04/27 A/Illinois/03/2009

GQ221827 2009/04/28 A/Idaho/02/2009

GQ323515 2009/04/30 A/Kentucky/05/2009

GQ254710 2009/05/01 A/Paris/2591/2009

GQ365668 2009/05/02 A/Brandenburg/19/2009

GQ251037 2009/05/03 A/Italy/05/2009

GQ323497 2009/05/04 A/Georgia/03/2009

GQ219779 2009/05/05 A/Poland/37/2009

GQ169381 2009/05/06 A/Thailand/104/2009

GQ402235 2009/05/07 A/Canada-QC/RV1759/2009

GQ227546 2009/05/08 A/Stockholm/29/2009

GQ179931 2009/05/10 A/Nonthaburi/104/2009

CY041760 2009/05/11 A/New York/3413/2009

GQ323575 2009/05/12 A/Texas/34/2009

GQ183619 2009/05/16 A/Beijing/01/2009

CY043229 2009/05/17 A/New York/3501/2009

GQ223445 2009/05/18 A/GuangzhouSB/01/2009

CY044066 2009/05/19 A/New York/3568/2009

GQ243754 2009/05/20 A/Philippines/2001/2009

GQ243762 2009/05/21 A/Victoria/2004/2009

GQ402242 2009/05/22 A/Canada-MB/RV1977/2009

GQ243750 2009/05/23 A/Philippines/2009/2009

GQ402241 2009/05/24 A/Canada-MB/RV1975/2009

CY043277 2009/05/25 A/New York/3651/2009

GQ283487 2009/05/26 A/Finland/554/2009

GQ283492 2009/05/28 A/Finland/555/2009

CY044082 2009/05/29 A/New York/3715/2009

GQ360062 2009/05/30 A/Stockholm/33/2009

GQ293078 2009/05/31 A/Zhejiang/2/2009

GQ330646 2009/06/03 A/Moscow/IIV03/2009

CY043336 2009/06/04 A/Denmark/523/2009

GQ369275 2009/06/06 A/Stockholm/37/2009

GQ287620 2009/06/07 A/Fukuoka-C/1/2009

GQ365685 2009/06/08 A/Stockholm/44/2009

GQ365688 2009/06/09 A/Stockholm/39/2009

GQ351316 2009/06/11 A/Hong Kong/2369/2009

GQ463202 2009/06/13 A/Hunan/SWL3/2009

GQ392031 2009/06/17 A/Italy/127/2009

GQ494353 2009/06/20 A/Moscow/IIV05/2009

CY044149 2009/06/25 A/Bogota/0466N/2009

CY044157 2009/06/26 A/Managua/056N/2009

GQ463958 2009/06/29 A/Changsha/78/2009

GQ465696 2009/07/01 A/Canada-SK/RV2486/2009

GQ497278 2009/07/07 A/Poland/282/2009

GQ392023 2009/07/09 A/Moscow/IIV04/2009

GQ497279 2009/07/10 A/Poland/303/2009

GQ421204 2009/07/12 A/Ancona/05/2009

GQ499337 2009/07/14 A/Washington/28/2009

GQ496143 2009/07/22 A/Vladivostok/IIV17/2009

GQ499335 2009/07/28 A/Washington/29/2009
