# Supplementary material for: Modeling gene sequences over time in 2009 H1N1 Influenza A Virus populations
Source: Virol J. 2009 Dec 4;6:215. doi: 10.1186/1743-422X-6-215 (PMC2794274; doi:10.1186/1743-422X-6-215)
Supplement: Additional file 2 — FindModel results for NA genes of 2009 H1N1 IAV strains. A table describing the results found for different evolutionary models tested in this study. [file 1743-422X-6-215-S2.DOC]

**Additional File 2. FindModel Results for NA genes of 2009 H1N1 IAV strains.**

| **Model Name** | **Akaike Information Criteria** | **Log Likelihood** |
| --- | --- | --- |
| Jukes-Cantor | 5263.412078 | -2631.706039 |
| Jukes-Cantor plus Gamma | 5264.221584 | -2631.110792 |
| Felsenstein 1981 | 5214.282804 | -2604.141402 |
| Felsenstein 1981 plus Gamma | 5215.115102 | -2603.557551 |
| Kimura-two parameter | 5183.006082 | -2590.503141 |
| Kimura-two parameter plus Gamma | 5183.775584 | -2589.887792 |
| Hasegawa-Kishino-Yano | 5133.573776 | -2562.786888 |
| Hasegawa-Kishino-Yano plus Gamma | 5134.468378 | -2562.234189 |
| Tamura-Nei | 5134.162338 | -2562.081169 |
| Tamura-Nei plus Gamma | 5135.048582 | -2561.524291 |
| **General Time Reversible** | **5128.969962** | **-2556.484951** |
| General Time Reversible plus Gamma | 5129.885880 | -2555.942940 |
